# Recruiting and Collecting Data From Home Health Care Providers: Experiences and Lessons

**DOI:** 10.1093/geroni/igaf122.988

**Published:** 2025-12-31

**Authors:** Chenjuan Ma, Hillary Dutton, Sora Aikawa, Ann Lee, Jeanmarie Moorehead, Jenna Blind

**Affiliations:** New York University, New York, New York, United States; New York University, New York, New York, United States; New York University, New York, New York, United States; 1199SEIU Training and Employment Funds, New York, New York, United States; New York University Langone Health, New York, New York, United States; New York University Langone Health, New York, New York, United States

## Abstract

As home health care (HHC) becomes increasingly pivotal in serving millions of homebound Americans, the need for empirical evidence to inform quality HHC delivery is imperative. However, conducting HHC research has unique challenges related to recruitment, data collection, and stakeholder engagement. Based on our experience conducting a study involving licensed HHC providers and HHC aides, this abstract presents key strategies for navigating these challenges to enhance research feasibility and quality. First, building partnerships with clinical and non-clinical HHC stakeholders facilitates access to participants. Identifying a dedicated site liaison, engaging them early in the research process, and incorporating their recommendations strengthens recruitment and fosters trust among participants. Fair and transparent compensation approaches that consider participant preferences incentivizes participation while maintaining ethical standards. While virtual approaches are feasible for data collection, they may require more structured facilitation to maintain smooth and meaningful interactions. Planning for potential technological challenges and providing anticipatory guidance ensures smoother execution of interviews. Flexibility in data collection methods—including focus groups and individual interviews—accommodates participant needs, and tailoring interview language based on participants’ professional backgrounds enhances response quality. While using technology for live transcription may expedite data processing, manual quality checks remain essential for accuracy. Lastly, leveraging multilingual research team members broadens inclusivity and representation. By integrating these strategies, researchers can enhance the rigor of HHC studies, ensuring meaningful participant engagement while capturing the complexities of their experiences. Future research should continue refining these approaches to strengthen data collection and partnership efforts in HHC settings.

